# Effect of rapeseed oil aromatisation with marjoram on the content of volatile fraction and antioxidant properties

**DOI:** 10.1007/s13197-019-04149-y

**Published:** 2019-11-09

**Authors:** Radosław Kowalski, Grażyna Kowalska, Urszula Pankiewicz, Marzena Włodarczyk-Stasiak, Monika Sujka, Artur Mazurek

**Affiliations:** 1 Department of Analysis and Evaluation of Food Quality, University of Life Sciences in Lublin, 8 Skromna Street, 20-704 Lublin, Poland; 2 Department of Tourism and Recreation, University of Life Sciences in Lublin, 15 Akademicka Street, 20-950 Lublin, Poland

**Keywords:** Aromatisation, Rapeseed oil, Marjoram essential oil, Sonication, Microwave

## Abstract

The aim of the paper was to study how the process of aromatisation with marjoram affected the composition of volatile fraction and antioxidant properties of rapeseed oil. Different methods of aromatisation were used: direct addition of marjoram essential oil, classical macerations of marjoram herb, and maceration assisted with ultrasound or microwave. The dominant aromatic component in the volatile fraction was γ-terpinene with concentration in the range from 3.15 μg/mL (microwave assisted maceration) to 8.82 μg/mL (classic maceration with shaking). The content of this compound in the mixture of rapeseed oil with essential oil was 152.09 μg/mL. The sample aromatized by the direct addition of essential oil contained the highest amount of volatile substances but simultaneously it had the lowest antioxidant activity.

## Introduction

Aromatisation is the enrichment of products in flavour and aromatic compounds—comparatively strong-smelling organic substances with characteristic, mostly pleasant odours. Flavours are chemical substances sensed by cells of the olfactory epithelium and, to a lesser degree, by gustatory buds on the tongue (Bauer et al. 1990). The sense of smell is the most important in the sensing and perception of food aroma, and thus plays a great role in the sensory assessment of a product and consequently in the evaluation of its quality. It is a sense with high and versatile sensitivity, and therefore the consumer places it first among the traits responsible for the quality of a food product. The contemporary science on human nutrition presents the view that flavours should be treated as fundamental and significant components of food products (Tournier et al. 2007). Natural flavours, i.e. essential oils, find the most extensive application and are used among others as agents for food preservation. This is due to consumers’ concerns regarding the safety and the negative effects of the use of synthetic food additives (Bruni et al. 2004).

Essential oils of plants are soluble in fats and there can be extracted with their help. Suitably prepared herbal material is put directly into fat or placed in flax bags immersed in fat. The process can be accelerated by means of slow stirring. The absorbents used include vegetable oil, olive oil, a mix of tallow and lard. The time of maceration is selected experimentally. After the extraction the raw material is removed and replaced with a fresh batch, repeating the process of extraction several times. Extracted material is separated through filtering or centrifuging, and residues of oil are extracted using a press. Aromatic oils obtained in this manner are called pomades or flavoured oils (Assami et al. 2016).

Essential oils are characterised by a broad spectrum of biological activity, including antimicrobial activity, and display a positive effect in combinations with synthetic food preservatives, allowing a reduction of the concentration of synthetic compounds applied. Such a role is played not only by isolated essential oils, but also by herbs and spices that contain those natural components and are used in e.g. canned food and pickles. Some foods and dishes are inseparably related with characteristic spices (Brud 2000). The addition of spices enables to increase the range of products by diversifying their taste and smell characteristics.

Herbs as spice are an important source of antioxidants, and apart from essential oils, they contain also other components (phenolic compounds, saponins, carotenoids) that inhibit the oxidation of lipids (Turek and Stintzing 2013). The addition of spices to food has an extremely long history and has been related with pleasure, valued by the hedonists as far back as the ancient times (Srinivasan 2005). The beginnings of the use of spices go back to the times when it was discovered that characteristic components occurring in natural products (aromas) can be easily obtained. Methods allowing to isolate those substances through extraction with olive oil or distillation have survived from the since ancient times till now. However, the original function of most spices was not to impart taste and flavour but primarily to conserve food (Brud 2000). Herbal spices are used as bactericidal and fungicidal agents for the control of correct run of lactic fermentation e.g. in the process of cucumber pickling.

Aromatised oils are a spice product in the kitchen that has been known for ages. Depending on the kind or aroma, aromatised oils can be added to meat dishes, salads, soups, sauces, and also to confectionery products (Ayadi et al. 2009). An alternative application of aromatised oils can be diet supplementation, where such products can be a source of health-promoting components for the organism. In addition, aromatised oils can be used in cosmetics. Aromatized oils can be obtained by classical maceration or with the use of ultrasound- or microwave-assisted methods. Previously published data indicate that the use of the latter methods can significantly accelerate the obtainment of the end product (Paduano et al. 2014).

The fact that aromatised oils are obtained through various physical processes was the impulse for undertaking a study in this area. Therefore the objective of this study was to estimate the effect of aromatisation of rapeseed oil with marjoram *Origanum majorana* on the content of volatile substances and to analyse the antioxidant properties of the aromatised oils obtained. The presented work is a continuation of research on fat aromatization with various raw plant materials (Kowalski et al. 2018).

## Materials and methods

The experimental part was developed according to the procedure previously described (Kowalski et al. 2018).

### Experimental material

The research material was constituted of the following: marjoram herb acquired from plant material from the herbal company Herbost (Kębłów, n. Piaski); rapeseed oil Kujawski (from so-called first cold pressing), ZT Kruszwica S.A. batch No. 15:33k.

### Essential oil distillation

Hydrodistillation process (180 min) of marjoram essential oil was performed using a Deryng-type apparatus (Kowalski et al. 2018). The essential oil was collected in a vial and stored at below − 25 °C in the dark until tested and analysed. The distillation was performed in triplicate.

### Preparation of samples: aromatisation of oil

#### Classical macerations

Marjoram flavored rapeseed oils were prepared by maceration of the herb of marjoram with 10% concentration (w/v). Two samples of rapeseed oil (100 mL) were transferred to glass bottles. Next the dried herb of marjoram (10 g) was added to each bottles (in 3 replicates) and maceration was conducted: samples were taken at 60 min (samples A) and 300 min of maceration (samples B) with shaking (25 °C).

#### Ultrasound-assisted macerations

The samples of flavoured rapeseed oil were prepared similarly to classical maceration. Next the samples were subjected at 60 min (samples C) and 300 min of maceration (samples D) with sonication (ultrasound bath Sonic-6, Polsonic, Poland; ultrasound power, 240 W; frequency, 40 kHz, 25 °C (the maceration in one continuous cycle).

#### Microwave-assisted macerations

The samples of flavoured rapeseed oil were prepared similarly to classical maceration and subjected to microwave treatment (samples E) for 60 min (MARS 5, CEM Corporation, Matthews, NC, USA; frequency, 2450 MHz, 40 °C).

#### Mixture of rapeseed oil with marjoram essential oil

In addition mixture of rapeseed oil with marjoram essential oil was prepared (samples F)—rapeseed oil 100 mL/essential oil 120 μL (obtained from herb of marjoram in an amount equivalent to the content for 10 g of raw herbal material).

The macerates obtained (after filtering) and the mixture of rapeseed oil with marjoram essential oil were used directly for chromatographic analysis for the content of aromatic volatile substances. For that purpose, portions of 5 mL of the oil solutions were taken into a vial, adding 10 μL of a mixture of internal standards (dodecane, nonadecane). The codes of individual samples are explained in the description of Table 3.

#### Determination of fatty acids

The rapeseed oil samples were prepared in accordance to previously described procedures by Kowalski (2007). Fatty acids from studied oil were converted to the corresponding fatty acid methyl esters (FAMEs) according to the literature (Kowalski 2009).

The GC-FID analyses of FAMEs were carried out on a GC Varian 450 system (Varian, USA) equipped with the Select™ Biodiesel for FAME capillary column (30 m × 0.32 mm × 0.25 µm film thickness), carrier gas He 28 mL/min, injector was set to 250 °C. and the detector to 300 °C; split ratio 1:100; injection volume 5 μL. GC oven temperature was programmed from 200 °C for 10 min, then incremented by 3 °C/min to 240 °C, 240 °C for 5 min.

The percentage of fatty acids was presented assuming that the sum of peak areas for all identified constituents is 100% (method of internal normalisation).

### Determination of essential oil chemical composition

#### Gas chromatography operating conditions

The GC analyses was performed in triplicate according to procedures described previously (Kowalski et al. 2015).

The GC–MS analyses were performed on a Varian 4000 GC–MS/MS system (Varian, USA), using the VF-5 ms silica capillary column (30 m × 0.25 mm; 0.25 μm film thickness), with He as the carrier gas at the flowrate of 0.5 mL/min. GC oven temperature was linearly programmed from 50 to 250 °C at the rate of 4 °C/min and kept at 250 °C for 10 min. The injector was set to 250 °C and the detector to 200 °C, split ratio 1:50; injection volume 5 μL. Electron ionization mass spectra were acquired in scan mode in the m/z range 40 to 870; EI mode, with ionization voltage 70 eV.

The GC-FID analyses were carried out on a GC Varian 3800 system (Varian, USA) equipped with the DB-5 fused silica capillary column (30 m × 0.25 mm × 0.25 µm film thickness), carrier gas He 0.5 mL/min, injector and detector FID temperatures 260 °C; split ratio 1:100; injection volume 5 μL. The GC program was the same as those used for GC–MS analysis.

### Headspace-gas chromatography operating conditions

The volatile fraction of the essential oils and the fat macerates (flavoured oils) were determined by the headspace-gas chromatography method. The headspace sampler was a pressure-loop HT3 model (Teledyne Tekmar, USA). The HS parameters used were: GC cycle time 71.00 min, valve oven temperature 200 °C, transfer line temperature 200 °C, platen/sample temperature 150 °C, sample temperature equilibration time 10.00 min, mixing time 1.00 min, mixer stabilize time 0.50 min, vial pressure 68.9 kPa, pressurization time 2 min, pressure equilibration time 0.2 min, loop fill pressure 34.5 kPa, loop fill time 2 min and inject time 1 min. The loop volume used on the HT3 (injection volume into the GC) was 0.25 mL. The GC–MS analyses were performed on a Varian 450 GC with the type triple qadrupol Varian 320-MS (Varian, USA), using the VF-5 ms silica capillary column (30 m × 0.25 mm; 0.25 μm film thickness), with He as the carrier gas at the flowrate of 0.5 mL/min. GC oven temperature was linearly programmed from 50 to 250 °C at the rate of 4 °C/min and kept at 250 °C for 10 min. The injector was set to 250 °C and the detector to 200 °C, split ratio 1:50; injection volume 5 μL. Electron ionization mass spectra were acquired in scan mode in the m/z range 40 to 1000 with 0.8 s/scan; EI mode, with ionization voltage 70 eV.

### Qualitative and quantitative analysis

The components were identified by comparing linear retention indices (RI), their retention times (RT) and mass spectra with those obtained from the authentic samples and/or the MS library (Kowalski and Wawrzykowski 2009). The quantitative analysis (internal standard addition method—alkanes C12 and C19) were performed according to previously described procedures (Kowalski and Wawrzykowski 2009). In addition, the relative percentages of the separated compounds were calculated from integration of the peak areas in the GC chromatograms.

### Preparation of samples: estimation of antioxidant properties

The antioxidant properties was assessed using the DPPH· method, as described by Sánchez-Moreno et al. (1998). An aliquot 100 μL of the aromatised oils was added to 2 mL of methanol in a vials and solutions were stirred. Portions of 0.5 mL of the solutions were taken and 3.5 mL of DPPH solution was to added to each of them (conc. 0.025 mg/mL, with specific absorbance at 515 nm). Absorbance was measured after 30 min at 515 nm (spectrophotometer UV Carry 100, Varian). The antioxidant activity of the macerates was expressed as a percentage of quenched DPPH radical after incubation with analysed sample for 30 min. in relation to the initial absorbance of DPPH solution.

The antiradical activity of the macerates was expressed in Trolox equivalents [mg/100 mL] calculated by creating a standard curve of Trolox standards versus their absorbance.

### Statistical analysis

Data were analysed using one way ANOVA followed by Duncan’s test using the SAS statistical system (SAS Version 9.1, SAS Inst., Cary, N.C., U.S.A.). The significance of all tests was set at *p* ≤ 0.05.

## Results and discussion

### The content of essential oil and fatty acids composition

The content of essential oil in herb of marjoram was 1.19% v/w. Compilation of the results of chromatographic analysis of fatty acids of rapeseed oil Kujawski is given in Table 1.

**Table 1:** Chemical composition of individual components in marjoram essential oil

Compound	RI	Concentration			
		A		B	C
		mg/mL	%	%	%
α-Thujene	932	9.13 ± 0.25	1.02	1.13	0.53
α-Pinene	939	6.08 ± 0.20	0.68	0.64	0.25
Camphene	955	0.20 ± 0.01	0.02	tr.	0.01
Sabinene	976	49.39 ± 1.78	5.51	5.79	3.06
β-Pinene	982	3.11 ± 0.05	0.35	0.11	0.23
Myrcene	992	11.82 ± 0.49	1.32	1.68	0.89
α-Phellandrene	1006	1.86 ± 0.04	0.21	0.49	0.26
ρ-Menth-1(7),8-diene	1007	0.36 ± 0.01	0.04	tr.	0.02
α-Terpinene	1018	59.26 ± 2.39	6.61	6.69	4.03
ρ-Cymene	1025	9.44 ± 0.06	1.05	1.02	0.69
Limonene	1029	12.65 ± 0.34	1.41	1.70	0.83
β-Phellandrene	1030	17.07 ± 1.12	1.90	1.73	1.59
Z-β-Ocimene	1037	0.60 ± 0.18	0.07	tr.	0.05
E-β-Ocimene	1049	0.75 ± 0.16	0.08	tr.	0.13
γ-Terpinene	1059	92.24 ± 3.34	10.29	10.80	8.22
*cis*-Sabinene hydrate	1070	48.36 ± 1.14	5.39	5.10	4.55
Terpinolene	1090	22.06 ± 0.64	2.46	2.46	1.69
Linalool	1099	20.52 ± 1.98	2.29	2.37	2.73
*trans*-Sabinene hydrate	1102	197.16 ± 5.32	21.99	26.40	30.16
*cis*-ρ-Menth-2-en-1-ol	1123	15.82 ± 0.12	1.76	1.25	1.27
*trans*-ρ-Menth-2-en-1-ol	1141	8.55 ± 0.10	0.95	0.76	0.70
Camphor	1145	0.06 ± 0.10	0.01	tr.	tr.
Menthone	1162	0.77 ± 0.04	0.09	tr.	0.07
Pinocarvacrol	1169	0.38 ± 0.14	0.04	tr.	tr.
iso-Menthone	1163	0.073 ± 0.00	0.01	tr.	tr.
Borneol	1170	0.53 ± 0.12	0.06	tr.	0.04
Terpinen-4-ol	1179	157.88 ± 5.37	17.61	20.14	27.65
α-Terpineol	1191	52.46 ± 1.82	5.85	2.75	5.53
*cis*-Dihydro carvone	1196	0.37 ± 0.09	0.04	tr.	tr.
*cis*-Piperitol	1201	2.94 ± 0.43	0.33	0.12	0.26
Nerol	1232	tr.	tr.	tr.	0.11
*trans*-Sabinene hydrate acetate	1258	4.46 ± 0.56	0.50	0.30	tr.
Linalool acetate	1259	25.04 ± 2.03	2.79	1.52	1.74
Bornyl acetate	1290	0.46 ± 0.14	0.05	0.07	0.02
Thymol	1294	0.94 ± 0.76	0.10	0.06	0.12
Menthyl acetate	1300	0.24 ± 0.16	0.03	tr.	tr.
Carvacrol	1304	1.15 ± 0.37	0.13	0.09	0.11
Terpinen-4-ol acetate	1305	5.99 ± 1.54	0.67	0.44	0.33
δ-Elemene	1341	0.41 ± 0.04	0.05	0.05	tr.
α-Terpinyl acetate	1351	0.13 ± 0.01	0.01	tr.	0.01
Neryl acetate	1364	0.66 ± 0.31	0.07	tr.	0.11
Geranyl acetate	1385	1.40 ± 0.59	0.16	tr.	0.22
E-Caryophyllene	1422	27.62 ± 1.33	3.08	2.32	1.14
Aromadendrene	1444	0.53 ± 0.05	0.06	0.05	tr.
α-Humulene	1458	1.30 ± 0.11	0.15	0.09	0.04
allo-Alomadendrene	1463	0.12 ± 0.00	0.01	tr.	tr.
Viridiflorene	1501	0.54 ± 0.05	0.06	tr.	tr.
Bicyclogermacrene	1505	14.50 ± 0.94	1.62	1.18	0.39
δ-Amorphene	1516	0.14 ± 0.00	0.02	tr.	tr.
γ-Cadinene	1518	0.06 ± 0.00	0.01	tr.	tr.
Spathulenol	1582	4.53 ± 0.80	0.51	0.46	0.10
Caryophyllene oxide	1588	2.86 ± 0.84	0.32	0.14	0.07
Globulol	1596	0.75 ± 0.07	0.08	tr.	0.01
Viridiflorol	1597	0.26 ± 0.15	0.03	tr.	tr.
Humulene epoxide II	1614	0.26 ± 0.01	0.03	tr.	tr.
epoxy-allo-Alloaromadendren	1645	0.27 ± 0.05	0.03	tr.	tr.

*RI* retention indices

A—gas chromatograph Varian GC 3800 with mass detector Varian 4000 MS/MS

B—gas chromatograph Varian GC 3800 with FID detector

C—gas chromatograph Varian GC 450 with Headspace and with mass detector Varian 320-MS

tr.—less than 0.05 mg/mL and 0.01%

**Fig. 1:**
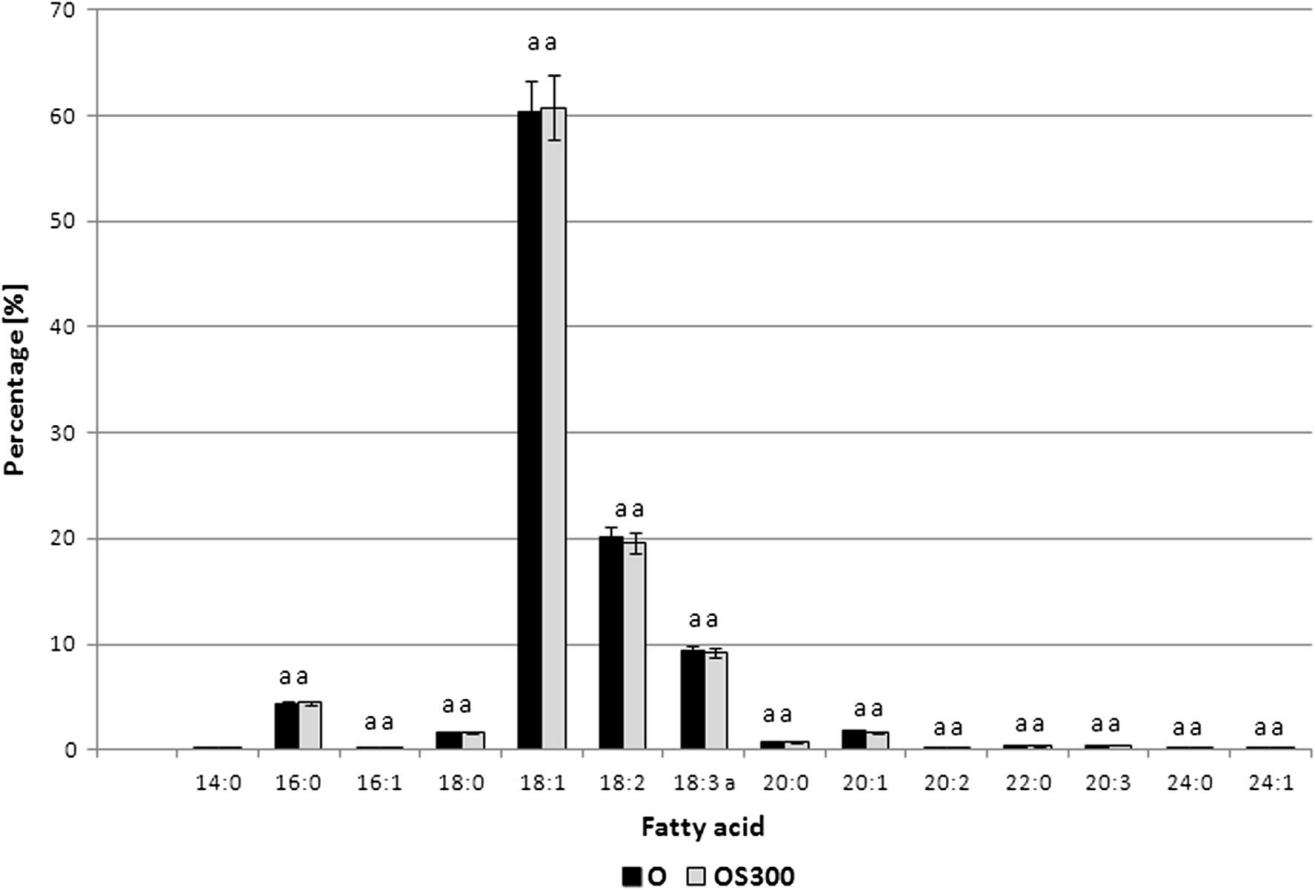
Fatty acid composition of rapeseed oil Kujawski (Kowalski et al. 2018). O—rapeseed oil Kujawski, OS300—rapeseed oil Kujawski subjected to sonication for 300 min, a, b, c, d…—values designated with the same letters do not significantly differ at 5% error (Duncan’s test)

In chromatographic analysis 14 fatty acids were identified (Fig. 1). The dominant fatty acid in the analysed fraction was oleic acid with a content of above 60% (60.28 ± 0.39). Among the other main fatty acids of the rapeseed oil we should include linoleic acid, with content of ca. 20%, and α-linolenic acid with content of ca. 9%. The results obtained are in conformance with literature data: C_18:1_—63.57%, C_18:2_—17.45%, C_18:3_—9.34%, C_16:0_—4.49% (Sigier et al. 2005). The composition of fatty acids is characteristic for cold-pressed low-erucic rapeseed oil. Rapeseed oil sonication for 300 min. did not have any significant effect on the composition of fatty acids. Application of other physical factors (temperature, microwaves) had no effect on the fatty acid profile of the rapeseed oil.

### The essential oil chemical composition

Table 1 present the results of GC chromatography analyses of marjoram oil. Essential oil obtained from marjoram through distillation displays traits intermediate between the terpineol chemotype (high content of terpinen-4-ol and the accompanying γ-terpinene, α-terpinene and α-terpineol) and the sabinol chemotype, with predominant occurrence of *cis*-sabinene hydrate and *trans*-sabinene hydrate and sabinene (Lis et al. 2007).

Chromatographic analysis of marjoram oil revealed the presence of 56 identified compounds (Table 1). The highest concentrations in the analysed essential oil were characteristic of *trans*-sabinene hydrate—197.16 mg/mL (ca. 22%) and terpinen-4-ol (157.88 mg/mL, 17.61%). Components with significant concentrations in the volatile fraction included also γ-terpinene (92.24 mg/mL, 10.29%), α-terpinene (59.26 mg/mL, 6.61%), α-terpineol (52.46 mg/mL, 5.85%), sabinene (49.39 mg/mL, 5.51%) and *cis*-sabinene hydrate (48.36 mg/mL, 5.39%).

### The content of volatile substances in the aromatised oils

**Fig. 2:**
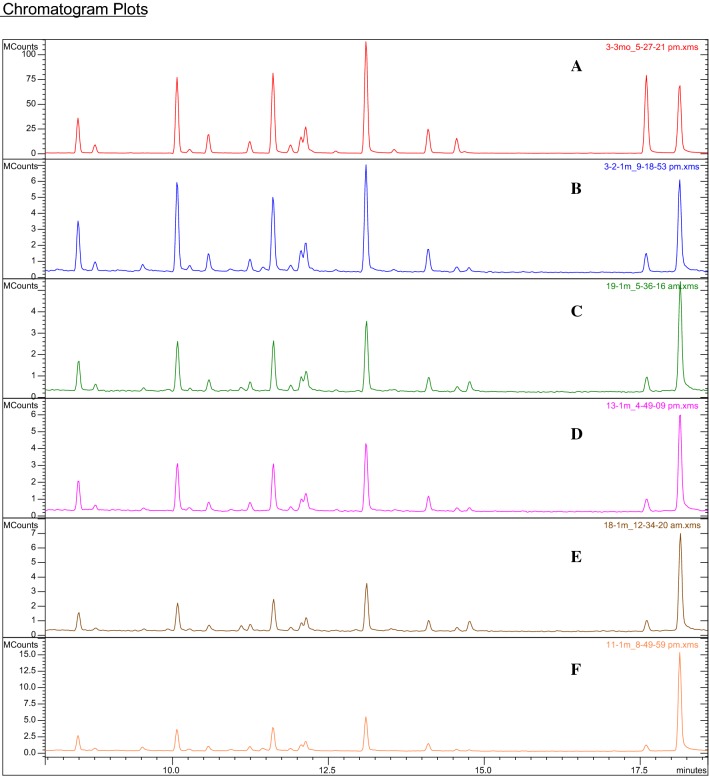
Chromatograms (GC/MS) of the volatile compounds found in flavoured rapeseed oil. A—macerate subjected to shaking for 60 min at 25 °C, B—macerate subjected to shaking for 300 min at 25 °C, C—macerate subjected to sonication for 60 min at 25 °C, D—macerate subjected to sonication for 300 min at 25 °C, E—macerate subjected to the effect of microwaves for 60 min at 40 °C, Frapeseed oil with an addition of essential oil

Table 2 present the results of GC analysis of the volatile fraction in rapeseed oil aromatised with herb of marjoram *Origanum majorana.* Chromatographic analysis of aromatised oils obtained experimentally (maceration) revealed the presence of 17 identified volatile components from the essential oil, while for the system prepared through direct addition of essential oil to rapeseed oil 21 substances were identified (Table 2, Fig. 2). The dominant aromatic component was γ-terpinene with concentration varied within the range from 3.15 μg/mL in macerate E (microwaves, 60 min, 40 °C) to 8.82 μg/mL in macerate B (classic maceration with shaking, 300 min, 25 °C), while in variant F (mixture of oil with essential oil) the content of γ-terpinene was 152.09 μg/mL. Terpinen-4-ol constituted the highest concentration in variant F (mixture of oil with essential oil), with a level of 205.75 μg/mL, while the level of that component in the macerates was notably lower: from 1.29 to 3.73 μg/mL. Moreover, the main aromatic components of the macerates occurred in the individual variants at the following levels of concentration: α-terpinene—from 1.89 to 5.13 μg/mL (for comparison, in oil F—88.05 μg/mL), sabinene—from 1.47 to 4.86 μg/mL (in oil F—71.74 μg/mL), and terpinolene—from 0.79 to 2.12 μg/mL (in oil F—35.76 μg/mL). In addition, the main aromatising components included also β-phellandrene and limonene. Statistical analysis of the results revealed that there were no significant quantitative differences for the main aromatised components of macerates obtained in different ways. Significant quantitative differences were found between macerates and oil aromatised through direct addition of essential oil.

**Table 2:** Concentrations of individual components of volatile fraction of aromatised rapeseed oil

Compound	RI	Concentration											
		F		A		B		C		D		E	
		mg/mL	%	mg/mL	%	mg/mL	%	mg/mL	%	mg/mL	%	mg/mL	%
α-Thujene	932	26.65 ± 0.39a	3.55	2.40 ± 1.35b	8.05	2.42 ± 1.05b	6.77	1.38 ± 0.84bc	7.07	2.53 ± 1.25b	7.41	0.96 ± 0.49c	7.73
α-Pinene	939	6.83 ± 0.28a	0.91	0.43 ± 0.23b	1.43	0.54 ± 0.26b	1.51	0.33 ± 0.30b	1.71	0.43 ± 0.32b	1.24	0.19 ± 0.10b	1.56
Sabinene	976	71.74 ± 0.54a	9.14	4.66 ± 2.68b	15.62	4.86 ± 2.08b	13.57	2.70 ± 1.90b	13.80	4.23 ± 2.49b	12.36	1.47 ± 0.76b	11.83
β-Pinene	982	3.29 ± 0.27a	0.44	0.24 ± 0.16bc	0.80	0.22 ± 0.11bc	0.61	tr.	tr.	0.37 ± 0.30b	1.07	0.10 ± 0.05c	0.79
Myrcene	992	18.86 ± 2.03a	2.51	0.91 ± 0.51b	3.05	1.16 ± 0.49b	3.25	0.49 ± 0.43b	2.51	1.17 ± 0.62b	3.42	0.32 ± 0.19b	2.58
α-Phellandrene	1006	11.19 ± 0.35a	1.49	0.69 ± 0.39bc	2.32	0.97 ± 0.39b	2.71	0.57 ± 0.47bc	2.90	1.12 ± 0.64b	3.28	0.34 ± 0.18c	2.75
α-Terpinene	1018	88.05 ± 1.45a	11.05	4.24 ± 2.41b	14.21	5.13 ± 2.09b	14.33	2.87 ± 1.96b	14.69	4.77 ± 2.28b	13.96	1.89 ± 0.98b	15.21
ρ-Cymene	1025	9.97 ± 1.20a	1.33	0.31 ± 0.16b	1.03	0.42 ± 0.20b	1.17	0.21 ± 0.11b	1.06	0.54 ± 0.27b	1.57	0.18 ± 0.12b	1.48
Limonene	1029	17.39 ± 1.99a	2.32	1.12 ± 0.66b	3.77	1.37 ± 0.59b	3.84	0.59 ± 0.50b	3.03	1.28 ± 0.68b	3.75	0.46 ± 0.23b	3.67
β-Phellandrene	1030	34.28 ± 1.07a	4.38	1.74 ± 1.10bc	5.83	2.30 ± 0.95b	6.43	1.13 ± 0.74bc	5.78	2.22 ± 1.14bc	6.50	0.73 ± 0.41c	5.87
Z-β-Ocimene	1037	2.86 ± 0.32a	0.38	tr.	tr.	0.44 ± 0.54b	1.22	tr.	tr.	tr.	tr.	tr.	tr.
E-β-Ocimene	1049	2.46 ± 0.17a	0.33	0.11 ± 0.05b	0.36	0.10 ± 0.07bc	0.28	tr.	tr.	tr.	tr.	0.02 ± 0.01 cd	0.14
γ-Terpinene	1059	152.09 ± 6.20a	19.06	7.01 ± 3.94b	23.53	8.82 ± 3.61b	24.64	4.92 ± 3.48b	25.16	8.19 ± 3.99b	23.97	3.15 ± 1.60b	25.30
*cis*-Sabinene hydrate	1070	4.84 ± 0.33a	0.64	tr.	tr.	tr.	tr.	tr.	tr.	tr.	tr.	tr.	tr.
Terpinolene	1090	35.76 ± 2.21a	4.76	1.80 ± 1.04b	6.06	2.12 ± 0.85b	5.92	1.07 ± 0.88b	5.50	2.06 ± 0.95b	6.04	0.79 ± 0.41b	6.32
Linalol	1099	20.8 ± 1.62a	2.77	0.44 ± 0.25b	1.46	0.56 ± 0.28b	1.56	0.32 ± 0.22b	1.66	0.67 ± 0.32b	1.96	0.18 ± 0.13b	1.47
*trans*-Sabinene hydrate	1102	2.33 ± 0.90a	0.31	tr.	tr.	tr.	tr.	tr.	tr.	tr.	tr.	tr.	tr.
Terpinen-4-ol	1179	205.75 ± 3.19a	26.95	3.02 ± 1.77b	10.13	3.54 ± 1.35b	9.89	2.40 ± 1.45b	12.28	3.73 ± 1.60b	10.92	1.29 ± 0.67b	10.34
α-Terpineol	1191	52.23 ± 1.60a	6.96	0.70 ± 0.41b	2.36	0.82 ± 0.32b	2.30	0.56 ± 0.34b	2.85	0.87 ± 0.37b	2.54	0.30 ± 0.16b	2.40
Geranyl acetate	1385	0.72 ± 0.10a	0.10	tr.	tr.	tr.	tr.	tr.	tr.	tr.	tr.	tr.	tr.
E-Caryophyllene	1422	4.69 ± 0.55a	0.62	tr.	tr.	tr.	tr.	tr.	tr.	tr.	tr.	0.07 ± 0.05b	tr.
Sum		772.78		29.81		35.79		19.54		34.18		12.44	

*RI* retention indices

A—macerate subjected to shaking for 60 min at 25 °C

B—macerate subjected to shaking for 300 min at 25 °C

C—macerate subjected to sonication for 60 min at 25 °C

D—macerate subjected to sonication for 300 min at 25 °C

E—macerate subjected to the effect of microwaves for 60 min at 40 °C

F—rapeseed oil with an addition of essential oil

tr—less than 0.05 mg/mL and 0.01%

a, b, c, d…—values designated with the same letters do not significantly differ at 5% error (Duncan’s test)

The total concentration of substances in the sample prepared through direct addition of essential oil to rapeseed oil was 772.78 μg/mL. Considerably lower concentration of volatile substances was observed in the macerates, which may result from the lower efficiency in extraction of volatile components using rapeseed oil compared to the yield of the distillation process. Aromatised oil obtained by shaking the raw material in oil—“B“, 300 min. at 25 °C—contained a total of 35.79 μg/mL of volatile components, while aromatised oil obtained with the use of sonication—“D“, 300 min at 25 °C—contained a similar level of flavours—34.18 μg/mL. Oils macerated for 60 min with classic shaking (“A“) as well as with sonication (“C“) had lower concentrations of aromatic compounds compared to macerations conducted for 300 min (“B“and “D“). The least effective method was that of maceration with the use of microwaves—“E“at 40 °C—12.44 μg/mL, however, in that oil a larger number of volatile compounds was observed—17. It can be assumed that the temperature of 40 °C (for the other systems in the experiment the temperature was 25 °C) during microwave maceration resulted in losses of components of the essential oil, which was observed phenomenon when using such assisted extraction (Ibrahim et al. 2012). The total amounts of aromatic compounds liberated from rapeseed oil in the course of analysis of the above-surface phase (headspace technique) were considerably lower that the concentrations that should be observed on the basis of calculations resulting from the addition of the raw materials and the essential oil. This fact may be related with various processes. Firstly, oil is a excellent carrier for aromatic substances, at the same time slowing down the release of volatile substances to the atmosphere—therefore components of essential oils can be bound is some manner with the fat system, which may be reflected in their retention and concentration of the volatile fraction. Secondly, in the process of maceration of herb of marjoram oil as a lipophilic medium liberates mainly components similar to that medium (the extraction process releases mainly lipophilic components). The extraction efficiency of maceration processes can be increased through by the choosing the right solvent or through modification of process parameters, e.g. extension of the time of maceration and/or increase of solvent temperature (Bucić-Kojić et al. 2009; Efthymiopoulos et al. 2018). However, referring to the so-called percentage share of aromatic components in the particular oil macerates one can conclude that it is fairly similar (similar distribution of components, while the absolute values, i.e. concentrations, display differences), which means that volatile compounds dissolve in oil with a similar trend, irrespective of the applied techniques of maceration. Comparing the percentage shares of volatile components in the macerates with oil aromatised through an addition of essential oil, especially notable differences were observed in the case of terpinen-4-ol, γ-terpinene, α-terpinene and sabinene.

Ultrasounds can effectively increase the efficiency of the process of extraction (Assami et al. 2016). Positive influence was also observed in ultrasound augmented distillation of essential oils, resulting in enhanced extraction of aromatic substances from plant secretory structures (Kowalski et al. 2015). Kowalski and Wawrzykowski (2009), and Da Porto and Decorti (2009) demonstrated that essential oils distilled from plant material in the presence of ultrasounds differed in their chemical composition from oils extracted with the classic method. Those authors attribute the changes to probable structural transformations of unstable chemical compounds under the effect of ultrasounds due to the production of nanoparticles (Taurozzi et al. 2011). In the experiment presented here no significant quantitative differences were observed, which may be related with the stabilisation of volatile aromatic compounds in the environment of rapeseed oil.

The application of microwaves in the distillation of essential oil has significant advantages in relation to the traditional solutions, and namely: shorter time of isolation (15 min compared to 180 min in the case of water distillation), enhanced antibacterial effect of the oil due to the formation of larger amounts of oxygen compounds. Under the effect of microwaves changes take place in the plant material, such as damage of cells and cell walls, destruction of secretory glands when some of them are filled with oil, which significantly accelerates the process of distillation. The observed changes are related with strains caused by rapid and violent evaporation of water, which causes the cracking and destruction of cell wall (Bousbia et al. 2009). However, the results obtained for macerate are not so satisfactory, as the lowest concentration of the substance was found in the oil solution, which may be the result of the influence of microwaves on the volatility of compounds (increasing the release of fragrances from the oil matrix). It may also be a consequence of increasing temperature to 40 °C (compared to 25 °C).

It can be concluded that microwave maceration was characterised by the lowest efficiency, while classic maceration and maceration with sonication were characterised by relatively similar levels of efficiency. Taking into account the economic costs of the somication and microwave processes, one should consider whether such small quantitative differences may have a bearing on the final profitability of the process.

The available literature lacks reports within similar areas, therefore it is difficult to compare the results obtained with literature data.

Previously published results of the study on aromatization of oil with rosemary are generally consistent with the results presented above (Kowalski et al. 2018). Rosemary and marjoram belong to the same botanical family (lamiaceae) and are characterized by a similar anatomical structure and the occurrence of similar secretory structures. On this basis, it can be concluded that the release of aromatic substances to the oil is also similar. At this point, the question arises whether similar relationships could exist for botanically and anatomically differentiated plant materials?

### Antioxidant properties of the aromatised oils

**Fig. 3:**
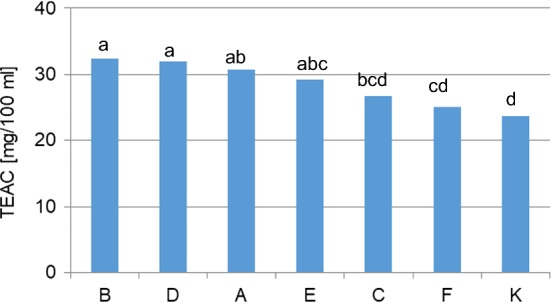
Antiradical activity of the macerates towards DPPH radicals expressed in Trolox equivalents (TEAC, Trolox Equivalent Antioxidant Capacity). A—macerate subjected to shaking for 60 min at 25 °C, B—macerate subjected to shaking for 300 min at 25 °C, C—macerate subjected to sonication for 60 min at 25 °C, D—macerate subjected to sonication for 300 min at 25 °C, E—macerate subjected to the effect of microwaves for 60  min at 40 °C, F—rapeseed oil with an addition of essential oil, K—control, rapeseed oil, a, b, c, d…—values designated with the same letters do not significantly differ at 5% error (Duncan’s test)

The analyzed macerates of rapeseed oil Kujawski with the marjoram herb were characterised by varied capacity for quenching DPPH radicals (Table 3 and Fig. 3). After 30 min the analysed macerates quenched from 26.21% (“C”) to 29.33% (“B”) DPPH. Macerates obtained by shaking were characterised by stronger antioxidant properties than macerates subjected top sonication, deactivating from 28.40% (“A”, maceration for 60 min) to 29.33% (“B”, maceration for 300 min) of the initial amount of DPPH radical (with sonication—26.21%—“C” after 60 min and 29.13%—“D” after 300 min, respectively). In the case of macerates subjected to sonication the capacity for radical neutralisation was at the level of 27.57% (“E”). The lowest capacity for quenching DPPH radical was characteristic of the sample prepared by direct addition of marjoram oil to rapeseed oil—“F” (25.21%). In the study 6 different variants were analysed. The difference between the macerate with the highest capacity for DPPH deactivation, compared with the material that reduced the radical to the least degree, was 4.12%. Converted to TEAC [mg/100 mL], the results can be arranged in a sequence in terms of antioxidant activity as follows (Fig. 1): macerate 300 min., shaking (32.36 ± 0.82), 300 min., sonication (32.01 ± 2.01), 60 min., shaking (30.70 ± 2.03), 60 min., microwaves (29.21 ± 3.09), 60 min., sonication (26.76 ± 2.96), direct addition of essential oil to oil (24.99 ± 2.69).

**Table 3:** DPPH radical quenching by the analysed macerates

Sample	DPPH radical quenching (%)
B	29.33 ± 0.46a
D	29.13 ± 1.12a
A	28.40 ± 1.27ab
E	27.57 ± 1.73abc
C	26.21 ± 1.65bcd
F	25.21 ± 1.50 cd
K	24.50 ± 0.52d

A—macerate subjected to shaking for 60 min at 25 °C

B—macerate subjected to shaking for 300 min at 25 °C

C—macerate subjected to sonication for 60 min at 25 °C

D—macerate subjected to sonication for 300 min at 25 °C

E—macerate subjected to the effect of microwaves for 60 min at 40 °C

F—rapeseed oil with an addition of essential oil

K—control, rapeseed oil

a, b, c, d…—values designated with the same letters do not significantly differ at 5% error (Duncan’s test)

Oxidation of lipids is a complex process that can be generated in three different ways: (1) with the participation of free radicals on the pathway of non-enzymatic chain reactions, (2) on the pathway of photo-oxidation (non-enzymatic, with no participation of free radicals), (3) on the pathway of enzymatic reactions (Miguel 2010). Among factors that can slow down the reaction of oxidation one can include reduced temperature, reduced oxygen pressure, inert gas environment, and also the presence of antioxidants (Kamal-Eldin and Pokorný 2005). An interesting group of compounds with antioxidant properties are essential oils (Miguel 2010), among which marjoram oil can have an application as an additive stabilising the composition of edible fats (Ezzeddine Nejla and Moncef 2006). The best known components of essential oils with antioxidant properties are phenolic compounds (thymol, carvacrol), however, the oils contain chemically diversified components, and some of them can even have a pro-oxidant effect (Amorati et al. 2013). Obviously, when analysing those natural mixtures, one should take into account their complex composition and the synergistic effect of the entire complex of chemical compounds (Amorati et al. 2013). Earlier research (Miguel 2010) indicates that volatile components with documented antioxidant activity include: α-terpineol, terpinen-4-ol, γ-terpinene, α-terpinene, α-thujene and sabinene, the presence of which was confirmed in marjoram-aromatised oils in the present study. In spite of the highest concentration of volatile compounds in the sample aromatised by direct addition of essential oil, that variant was characterised by the lowest antioxidant activity. Apart from essential oil, the herb of marjoram contains also other active components, i.e. diterpenes (carnosic acid, carnosol), triterpenes (ursolic acid and oleanolic acid), sterols, ether derivatives (monomethyl hydroquinone ether, arbutins), flavonoids (apigenins, hispidulins, luteolin-7-O-beta-glucoside, 6-hydroxyapigenin, 7-O-β-glucosides), that migrate to the solution during maceration (Vagi et al. 2005; Fecka and Turek 2008). Probably, it is the components mentioned above, occurring in marjoram and not the essential oil as such, that are responsible for the higher capacity for quenching DPPH radicals in macerates compared to the sample prepared through the addition of marjoram oil to rapeseed oil. Clodoveo et al. (2016) observed higher concentrations of polyphenol compounds and increased antiradical activity in aromatised olive oil by the use of ultrasound. In contrast, in our work, ultrasounds did not increase antiradical activity compared to classical maceration. Paduano et al. (2014) showed that the ultrasound-assisted extraction of capsaicinoids from chili peppers (*Capsicum annuum* L.) into flavored olive oil did not increase the antioxidant properties of the product compared to classical extraction, while the use of microwave-assisted extraction made it possible to obtain extracts of significantly higher antioxidant activity compared to the control sample. A significant antioxidant activity compared to the control sample was displayed by the macerate subjected to the effect of microwaves (29.21 TEAC [mg/100 mL), at relatively low volatile substance content—12.44 mg/mL. This can be attributed to the degradation of cell structures and liberation of phenolic compounds under the effect of microwaves (Bousbia et al. 2009).

In addition, analysis of the control sample K revealed that pure rapeseed oil is also characterised by DPPH radical quenching capacity at the level of 24.50% (23.70 TEAC [mg/100 mL]), and the addition of herb improves that capacity only slightly. Seeds of oil-bearing plants contain such compounds as fatty acids, phenolics, tocopherols, sterols, stanols, phospholipids, waxes, squalene and other hydrocarbons that migrate to oils during the process of cold pressing, due to which they can prevent oxidation caused free radicals (Yu et al. 2002). Canolol, present in rapeseed oil, being the product of decarboxylation of sinapinic acid, has strong antioxidant properties (Koski et al. 2002). The study of antioxidant activity of rapeseed oil conducted by Sigier et al. (2008) demonstrated reduction of DPPH radical at the level of 50%. Values of DPPH radical reduction obtained in this experiment, lower by half (24.50%), can be attributed to varietal, natural and technological factors that may affect the final chemical composition of oils. Antioxidant activity of the analysed samples is relatively low (ca. 28% depending on the method of maceration) compared to literature data, e.g. for fruit tea extracts—ca. 64% of quenched DPPH radicals after 10 min (Szlachta and Małecka 2008), while the antioxidant activity of the experimental aromatised oils is higher than the activity of such dry spices as pepper, parsley tops or caraway (ca.18%) (Gawlik-Dziki and Świeca 2007). Whereas the DPPH radical scavenging activity of oils aromatised with rosemary under similar experimental conditions was higher, reaching the following maximum values: 30.31% (sonication for 60 min at 25 °C) and 31.89% (sonication for 300 min at 25 °C) (Kowalski et al. 2018). Differences between aromatised oils obtained in parallel experiments, differing in addition to the vegetable raw material, probably result from their chemical characteristics. Chiang Chan et al. (2012) showed that fresh rosemary herb was characterized by a higher antioxidant activity compared to marjoram herb, which is confirmed by the results of the presented work and the results published earlier (Kowalski et al. 2018).

Studies show that the fat aromatization method affects the content of volatile compounds, the concentration of which in turn does not necessarily involve an increase in antioxidant properties. The simplest and most effective way of oil aromatization is a direct addition of essential oil, however our study showed that the obtained product did not have the highest antioxidant activity. Whereas classical methods of aromatization (maceration) or alternative methods with ultrasonic or microwave support may lead to a product with better antioxidant properties. Another aspect that requires further comprehensive research is chemical and morphological diversity of plant materials.

## Conclusion

1. The study showed that the method of aromatisation affects the content of volatile substances.
2. It was demonstrated that direct addition of essential oil to oil is the most effective method of aromatisation in terms of the content of volatile components.
3. It was demonstrated that the time of extraction and sonication affect the efficiency of the process of aromatisation.
4. It was found that the antioxidant activity of aromatised oils did not depend on the concentration of volatile compounds in the oil.
5. It was demonstrated that oils aromatised through maceration of plant material with oil were characterised by higher antioxidant activity, which was most likely related with the presence of active substances other than volatile compounds.
